# Influenza Forecasting with Google Flu Trends

**DOI:** 10.1371/journal.pone.0056176

**Published:** 2013-02-14

**Authors:** Andrea Freyer Dugas, Mehdi Jalalpour, Yulia Gel, Scott Levin, Fred Torcaso, Takeru Igusa, Richard E. Rothman

**Affiliations:** 1 Department of Emergency Medicine, Johns Hopkins University, Baltimore, Maryland, United States of America; 2 Whiting School of Engineering, Johns Hopkins University, Baltimore, Maryland, United States of America; 3 University of Waterloo, Waterloo, Ontario, Canada; National Institutes of Health, United States of America

## Abstract

**Background:**

We developed a practical influenza forecast model based on real-time, geographically focused, and easy to access data, designed to provide individual medical centers with advanced warning of the expected number of influenza cases, thus allowing for sufficient time to implement interventions. Secondly, we evaluated the effects of incorporating a real-time influenza surveillance system, Google Flu Trends, and meteorological and temporal information on forecast accuracy.

**Methods:**

Forecast models designed to predict one week in advance were developed from weekly counts of confirmed influenza cases over seven seasons (2004–2011) divided into seven training and out-of-sample verification sets. Forecasting procedures using classical Box-Jenkins, generalized linear models (GLM), and generalized linear autoregressive moving average (GARMA) methods were employed to develop the final model and assess the relative contribution of external variables such as, Google Flu Trends, meteorological data, and temporal information.

**Results:**

A GARMA(3,0) forecast model with Negative Binomial distribution integrating Google Flu Trends information provided the most accurate influenza case predictions. The model, on the average, predicts weekly influenza cases during 7 out-of-sample outbreaks within 7 cases for 83% of estimates. Google Flu Trend data was the only source of external information to provide statistically significant forecast improvements over the base model in four of the seven out-of-sample verification sets. Overall, the p-value of adding this external information to the model is 0.0005. The other exogenous variables did not yield a statistically significant improvement in any of the verification sets.

**Conclusions:**

Integer-valued autoregression of influenza cases provides a strong base forecast model, which is enhanced by the addition of Google Flu Trends confirming the predictive capabilities of search query based syndromic surveillance. This accessible and flexible forecast model can be used by individual medical centers to provide advanced warning of future influenza cases.

## Introduction

Influenza is a substantial cause of morbidity and mortality with up to five million cases of severe illness and 500,000 deaths worldwide each year [1]. In the United States, seasonal influenza results in increased emergency department (ED) visits and hospitalizations, straining an already stressed medical system [2], [3], [4], [5], [6]. Increased patient volume caused by seasonal influenza is a contributor to ED crowding, which has been linked to delays in critical treatments and increased mortality [7], [8], [9], [10]. An influenza pandemic presents a well recognized and serious threat to the United States healthcare infrastructure [3], [6]. Effective management of both seasonal and pandemic influenza requires early detection of the outbreak through timely and accurate surveillance linked with a rapid response to mitigate crowding.

Numerous potential surveillance systems exist to identify influenza outbreaks. Traditional surveillance such as The Centers for Disease Control and Prevention’s (CDC) US Influenza Sentinel Provider Surveillance Network relies on the collection of numerous indicators including clinical symptoms, virology laboratory results, hospital admissions and mortality statistics resulting in a several week lag in data reporting [11]. New digital surveillance sources, such as Google Flu Trends (GFT), offer the potential to identify influenza surges in real-time, optimizing timely outbreak detection and response. GFT utilizes internet search queries to detect the presence of influenza like illness (ILI) on a national, regional, state and city level 7–10 days prior to the U.S. Influenza Sentinel Provider Surveillance Network and was recently validated to show a strong correlation with ED influenza cases at a local level [12], [13], [14]. However, the forecasting capabilities of GFT remain unknown. Given the real-time nature of GFT surveillance, and the demonstrated strong correlation of GFT with ED influenza cases, GFT has the potential to go beyond early detection and forecast future influenza outbreaks.

Previous forecast models have lacked flexibility, due to restrictive or inappropriate assumptions, technically demanding computational requirements, or inclusion of data elements which are not universally available in real time, reducing practical utility. Initial influenza prediction models followed the classic compartmental Susceptible-Infected-Recovered (SIR) or Susceptible-Exposed-Infected-Recovered (SEIR) framework [15], [16], [17]. Model parameters representing flow between compartments require frequent parameter refitting in order to track and update. Others have relied on the nonparametric method of analogues, which is rooted in meteorology, and based on selecting historical patterns of influenza dynamics that most closely match current influenza observations for forecasting future influenza outbreaks [18], [19], [20]. As mentioned by Ackerman and Knox, the method of analogues approach is not suitable for practical implementation, due to a virtual impossibility of selecting a perfectly matching analog, as well as the sensitivity of the forecasts to minor mismatches in selected patterns [21]. Recent suggestions to forecast influenza outbreaks employ either the particle learning approach coupled with Bayes Factors, or a chain binomial model [22], [23]. Though both may provide accurate predictions, they involve computationally intensive routines of parameter estimation limiting their practical applicability in the clinical setting.

Several recent influenza forecasting studies have used a Box-Jenkins methodology, in particular, an autoregressive integrated moving average (ARIMA) model [24], [25], [26]. These models assume Gaussian errors for residuals and can be applied to count data using a logarithmic transformation. In order to capture the most recent outcomes in influenza counts and to assess non-stationarity of influenza dynamics, we chose to employ the Generalized Autoregressive Moving Average (GARMA) model with discrete-valued distributions and integrated external variables [27]. External variables include any data used for prediction that is independent of the outcome predicted (i.e. weekly counts of influenza cases).

Published forecast models integrate the main outcome variable, typically previous influenza cases or ILI visits, with several external variables related to influenza surveillance (type of influenza virus, number of emergency medical service [EMS] calls, sick leaves, and over the counter drug sales) or climate (temperature, humidity, rainfall, and atmospheric pressure) to produce forecasts [19], [25], [26]. Several of these surveillance data elements are often not available in real-time which severely limits their usefulness for real-time prediction. However, meteological factors, which have previously shown significant correlation with influenza cases, are often available in real time in targeted geographic areas, and can be easily accessed via the internet [28], [29], [30].

Optimizing management of influenza outbreaks relies on early detection tied to a timely and effective response. This requirement is amplified in the ED where influenza-related crowding can impact quality of patient care. Our primary objective was thus to develop and validate a forecast model which would be practically useful and have broad applicability for providing advanced warning of an influenza outbreak. Accordingly, we chose to include only easily accessible, real-time data, available at the city or medical center level. Our secondary objective was to evaluate the added predictive capability of novel search query-based surveillance, such as GFT, in comparison and integrated with more commonly considered meteorological information.

## Methods

### Study Population and Setting

This is a retrospective evaluation of data from an urban tertiary care ED with an annual volume of 60,000 adult and 24,000 pediatric visits.

### Data Collection and Methods of Measurement

The primary outcome was number of influenza-related ED patient visits over seven influenza seasons from 2004 to 2011. Weekly influenza-related ED visits were calculated by summing the number of patients with a positive influenza test sent from the ED during each week of the study period. This study was approved by the institutional review board with a waiver of consent as this study used anonymous, aggregated data. Additional external sources of information including GFT, local temperature (degrees Fahrenheit), local relative humidity, and Julian weeks (week of the year from January 1, listed as week 1, to December 31, listed as week 52, and considering the leap years with 53 weeks) were examined for predictive capability. External data is publically available for download on a daily (i.e. real-time) basis. GFT data for the city of Baltimore was downloaded directly from http://www.google.org/flutrends in April 2012 [12]. Daily temperature and relative humidity measures were downloaded directly from Weather Underground for the city of Baltimore and then averaged over each week to correspond with Google Flu Trend and influenza case data [30].

### Statistical Analysis

Forecast models were developed from training sets and evaluated against out-of-sample verification sets using a leave-one-out approach. We partitioned the data set into 7 years, where each year begins near September 1, and includes 52 or 53 weeks depending on the leap year status. This left us with 5 typical influenza seasons (2004–2008 and 2010–2011) and 2 atypical influenza seasons (2008–2009 and 2009–2010). We then trained our model for 6 seasons and validated the model on the remaining season, and continued this approach until each season has been used in the validation set exactly once.

Models were compared using a summation of the global forecast deviance of each of the verification sets (further referred to as the global forecast deviance), as recommended for GARMA and GLM models [27], [31], and forecast confidence. Global deviance is a statistical measure of accuracy, which is defined as twice the negative of the log-likelihood function magnitude of the fitted model on the verification data set. Therefore, global forecast deviance measures the lack of fit between the fitted model and the actual forecasted data, and thus a model with lower global forecast deviance is preferred. Forecast confidence is the percentage of forecast values that are within a predefined difference of the actual data during an influenza peak (here chosen as 20% of the mean of the maximal point of the influenza peak, or seven influenza cases). For this evaluation, an influenza peak is defined as three or more weeks with three or greater confirmed influenza cases. Although forecast confidence provides an easily interpretable evaluation of the model’s performance, all model selection was based upon the more statistically rigorous global forecast deviance. Time series models that showed autocorrelation of residuals were discarded from the analysis.

The GARMA(p,q) model with Poisson or Negative Binomial distribution used for this analysis can be expressed as:
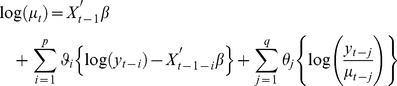
(1)


In Equation (1), primes stand for transpose, β, Φ and θ are model parameters, which were estimated based on data from the training set and a maximum likelihood approach, μ is expected value of response (y), and X is the vector of external variables. A logarithmic link function is used here as proposed by Benjamin *et al* for the case of Poisson or Negative Binomial distribution. The external variables are lagged by one-week with respect to forecasted values to ensure that the model uses only available data to for a prediction one week in the future [27]. Based on global forecast deviance, we chose to employ a Negative Binomial GARMA (3,0) model as inclusion of moving average terms or using a Poisson distribution yielded a higher global deviance. The Negative Binomial distribution outperforms the Poisson distribution because it adjusts for over dispersion with the dispersion parameter (k). Using global deviance, we found that for our dataset, the best dispersion parameter for the Negative Binomial distribution is 2.

Primarily, forecast models with the outcome of a point estimate of counts of weekly influenza-related ED patient visits were designed using a Negative Binomial Generalized Autoregressive Moving Average model (GARMA) [27]. Secondarily, external variables such as GFT, meteorological data (temperature, change in temperature, and relative humidity) and temporal variables (Julian weeks, and seasonality) were modeled individually with Negative Binomial generalized linear models (GLM), and were subsequently also added to the baseline model as external variables using a forward selection method. This univariate analysis was used to assess the predictive set of variables for the final forecast model. The developed MATLAB routine for modeling and forecasting influenza counts with GARMA models along with the user manual is publicly available [32].

## Results

**Figure 1:**
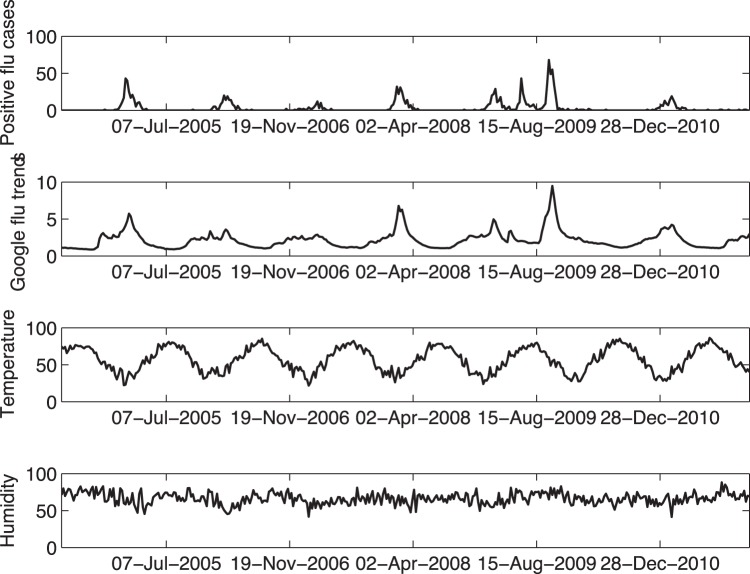
Input variables over study timeframe.

**Figure 2:**
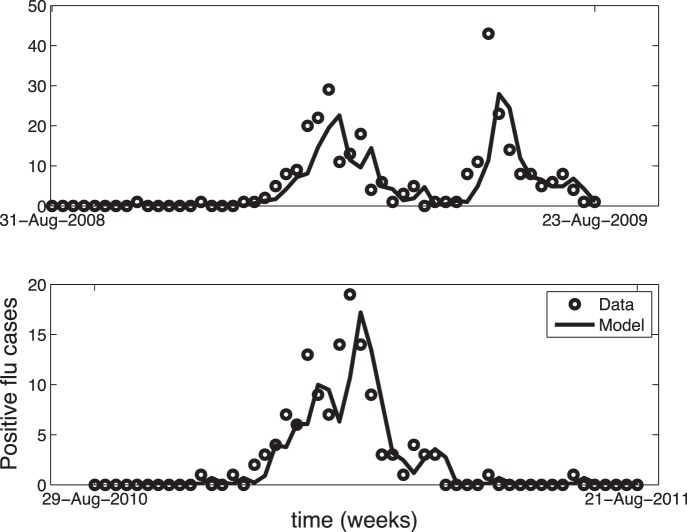
Base Autoregressive Forecast Model. Number of confirmed Emergency Department (ED) influenza cases (dots) compared to the base 3^rd^ order Negative Binomial Generalized Autoregressive Poisson (GARMA) model (line) over (a) the 2008–2009 atypical influenza season and (b) the 2010–2011 typical influenza season.

The derived forecast models are based upon seven influenza seasons of weekly data including number of influenza-related ED patient visits, GFT, mean temperature, and mean relative humidity as shown in Figure 1. The eight included influenza peaks (the 2008–2009 season had 2 peaks) spiked at a mean value of 33.1 (95% confidence interval: 20.5–45.5) weekly influenza cases. Several GLM and GARMA forecasting models were examined systematically to determine the optimal (most accurate) set of input data characterized by the final model, as shown in Table 1. As expected, the GARMA model displayed the lowest global forecast deviance (i.e., highest accuracy). GFT alone significantly outperformed temperature, relative humidity, and Julian weeks when using GLM models. Forecasts from this final model are shown in Figure 2 during both an atypical (2008–2009) and a typical (2010–2011) influenza season, where the quality of this model can be observed.

**Table 1:** Forecast of Emergency Department Influenza Cases.

Covariate	Forecast Global Deviance	Forecast Confidence
*Autoregression*		
First order autoregression	1049	81%
Second order autoregression	1039	79%
Third order autoregression	1016	81%
*Google Flu Trends*		
Google Flu Trends	1609	77%
Δ Google Flu Trends	2758	56%
*Climate*		
Temperature	2470	66%
Δ Temperature	3070	60%
Humidity	2181	69%
*Time*		
Julian weeks	2890	63%
Sin(2π/52) + Cos(2π/52)	3551	62%

Capability of Generalized Autoregressive Negative Binomial (GARMA) and univariate generalized linear models (GLM) to forecast the number of confirmed Emergency Department (ED) influenza cases. Δ indicates the change of the indicated variable between the prior and current week. Forecast Global Deviance indicates the sum of each forecast global deviance for all 7 leave-one-out validation models. Forecast Confidence indicates the average of confidences from all 7 leave-one-out validation models. Forecast confidence is the percentage of forecast values, during an influenza peak, that are within seven influenza cases of the actual data.

**Figure 3:**
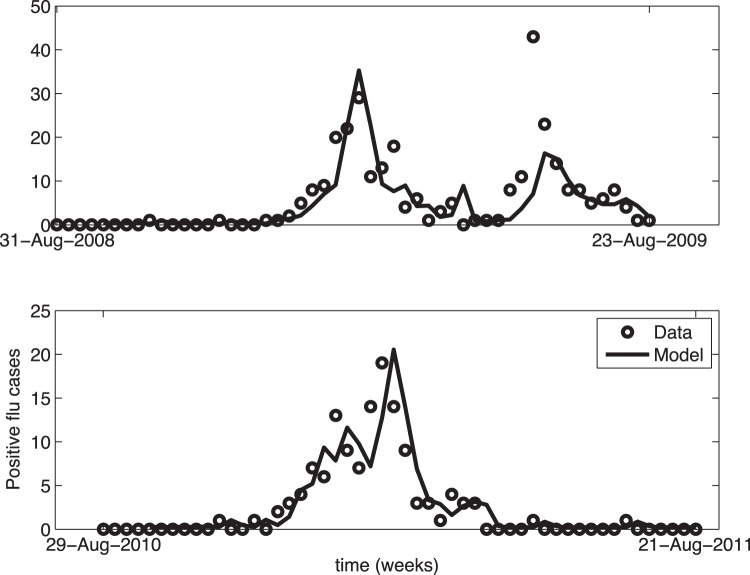
Final Autoregressive Forecast Model. Number of confirmed Emergency Department (ED) influenza cases (dots) compared to the final 3^rd^ order Negative Binomial Generalized Autoregressive Poisson (GARMA) model with Google Flu Trends as an added external variable (line) over (a) the 2008–2009 atypical influenza season and (b) the 2010–2011 typical influenza season.

Using the selected GARMA(3,0) base model, several external variables were added using a forward selection method and were evaluated for improved forecasting capabilities which are presented in Table 2. Adding GFT significantly improved the model as demonstrated by the highly statistically significant p-value of 0.0005. On all 7 out-of-sample verification sets, GFT was the only exogenous variable to be statistically significant at a significance level of 0.05 in 4 of the verification sets (seasons 2005–2006, 2006–2007, 2009–2010, and 2010–2011). No other variable was significant in any of the out-of-sample verification sets. Assuming the inclusion of GFT using forward selection, addition of any of the remaining external variables did not significantly reduce the global forecast deviance of the final model, or sometimes increased it due to over-fitting. Thus, the final model selected was GARMA(3,0) with Negative Binomial distribution and dispersion parameter of 2 with Google Flu Trend data as the external variable lagged back one week with respect to the responses. This model has a forecast confidence of 83% indicating that 83% of the forecasted values, during all influenza peaks, were within seven influenza cases of the actual data. As displayed in Figure 3, the forecast of this model indicates that the yielded GARMA out-of-sample forecasts closely follow the observed influenza counts during both an atypical (2008–2009) and typical (2010–2011) influenza season.

**Table 2:** Capability of adding an exogenous covariate to forecast.

Covariate	Forecast Global Deviance	Forecast Confidence	*P-value*
none (Baseline model)	1016	81%	
*Google Flu Trends*			
Google Flu Trends	1004	83%	0.0005
Δ Google Flu Trends	1017	80%	>0.05
*Climate*			
Temperature	1039	80%	>0.05
Δ Temperature	1017	83%	>0.05
Humidity	1030	82%	>0.05
*Time*			
Julian weeks	1014	82%	>0.05
Seasonality−Sin(2π/52)+Cos(2π/52)	1040	83%	>0.05

The number of confirmed Emergency Department (ED) influenza cases compared to the base 3^rd^ order Negative Binomial Generalized Autoregressive Poisson (GARMA) model. Δ indicates the change of the indicated variable between the prior and current week. Forecast Global Deviance indicates the sum of each forecast global deviance for all 7 leave-one-out validation models. Forecast Confidence indicates the average of confidences from all 7 leave-one-out validation models. Forecast confidence is the percentage of forecast values, during an influenza peak, that are within seven influenza cases of the actual data.

## Discussion

Seasonal and pandemic influenza leads to ED crowding, which results in reduced quality of patient care. Early detection or forecasting of an impending influenza outbreak, coupled with an effective intervention designed to mitigate ED crowding, allows for improved management of the anticipated increase in patient volumes. Though several surveillance systems have been designed to provide advance warning, few provide reliable data in near-real time, and fewer still have demonstrated the capability to provide advanced forecasting of impending influenza cases, which would provide the additional critical time necessary for activating a robust response.

Although hospital and ED planners often rely on information from their individual facilities, integration of broader surveillance information from the city, state, or national level can provide increased awareness and earlier detection of an impending threat [33], [34], [35], [36]. Previously, hospital and ED planners have used surveillance information to understand disease prevalence for testing and treatment decisions, institute infection control precautions to contain outbreaks, and anticipate ED surge [33], [34], [35], [36]. Particularly for anticipation or detection of ED surge, surveillance has lead to increased ED capacity and staffing, purchase of additional supplies, and reallocation of hospital resources such as staffing and beds [33], [36]. In a qualitative analysis by Buehler, one hospital planner noted “[The syndromic surveillance report] really helped me in continuity of business planning in reference to what we can anticipate in the next 24 hours, in reference to staffing our ERs and what our capacities were going to be” [33]. Earlier warning of an impending outbreak though a focused influenza forecast model could increase planning capabilities beyond simply the next 24 hours, giving hospitals the crucial time needed to prepare for increased patient volumes whether through distribution or purchase of supplies, increased staffing, or opening additional annex areas to increase bed capacity. An easily accessible, flexible, forecast model, such as the one developed here, could easily be distributed and geographically focused to provide individual medical centers with their own influenza prediction model, allowing for advanced influenza planning.

We sought to develop a practical forecast model, which could ultimately be used in a clinical setting to guide implementation of an intervention to mitigate crowding resulting from influenza. This end goal of clinical application limited the potential data sources which could be used in the forecasting model. Further, in order to be effective, the final model must not only be accurate and timely, but also easy to implement and applicable to both an individual city and clinical center. Thus, we selected data with these parameters in mind.

GFT can be narrowed down to city level data, is available in real time, and has been previously validated to strongly correlate with the number of influenza and ILI cases at the medical center level [14]. This particular surveillance system was chosen over others due to the near real-time nature of the data, which is available 7–10 days before traditional systems [13]. Additionally, GFT is a free data source, which is easily downloaded and available for all to access, permitting convenient input for potential end-users into a forecast model. These same principles of city level detail, real-time availability, and ease of use also applied to the other parameters we evaluated including meteorological variables and basic time variables. Notably, our model differs from many previous forecast models used for influenza surveillance which have incorporated parameters that are either difficult to access or not universally available in real-time, such as over the counter drug sales and school absenteeism [19], [25]. Inclusion of such variables in those models has limited their applicability with regard to data availability or feasibility of data collection and synthesis. The one element considered in this model which is not readily available from an online source is the number of influenza cases at the medical center where the model may be used; however, this data is almost always tracked and easily available to the medical institution itself.

As shown by the numerous models examined here, autoregression of actual influenza cases is critical to a strong influenza forecast model. Without the autoregressive component, models solely relying on surveillance, meteorological, or temporal components had much greater error in the resulting forecasts. Although GFT does statistically improve the baseline autoregressive forecast model, the practical significance of this improvement is marginal, as addition of GFT to the model improves the forecast confidence from 81% to 83%. However, given the ease of use and integration of GFT into the model, there is little downside to its inclusion in the final model. The significance of including confirmed influenza cases in the model underscores the importance of medical centers maintaining influenza testing programs, as well as efficient laboratory information systems which can process and result this data in a timely, accessible fashion.

Findings here build on prior work by our group where we reported a strong correlation between GFT and local influenza cases, and suggested an early rise in GFT 1–2 weeks prior to actual increases in confirmed influenza cases [14]. Our derived forecast model provides the first-ever demonstration of GFT’s forecasting capabilities by showing significant improvements in the forecasting estimates of a baseline GARMA model with the addition of GFT data. This validation of the predictive capabilities of GFT was performed using the current GFT algorithm. Previous GFT algorithms (prior to 2009) had lower correlation with influenza cases during the 2009 pandemic, likely due to a change in internet search patterns [37], [38]. Though the current algorithm is shown to have predictive capacities, there remains a future possibility of additional alterations to search patterns, and poor correlation between GFT and influenza cases requiring additional updates.

This study is limited by the lack of demonstration of regional generalizability, as the models were developed using influenza data from one medical center. Further, though the models used city-level GFT and meteorological data, it is unclear whether these data elements are uniform throughout the city or vary by smaller geographic regions. The ultimate forecast model should be flexible based on geographic area and local data; thus additional study is necessary to fully evaluate the geographic generalizability of our model. Temporal generalizability is also of concern; however, these models were validated using both atypical and typical influenza seasons, thus accounting for various potential influenza patterns.

Overall, we have developed a practical, city-based forecast model based upon generalized autoregression of laboratory confirmed influenza cases and GFT. The addition of real-time, easily accessible GFT improves the forecasting capabilities and demonstrates the predictive potential of GFT. This practical forecast model could provide advanced warning for medical centers, thus allowing time to implement an appropriate response to control infection or mitigate influenza-related increases in patient volumes. Ultimately designed for clinical application, integrating this potentially powerful forecasting tool into medical center use requires additional clinical feasibility and effectiveness studies to link this forecast with a clinical intervention designed to mitigate crowding.
